# Supplementary material for: Single-cell RNA sequencing of human nail unit defines RSPO4 onychofibroblasts and SPINK6 nail epithelium
Source: Commun Biol. 2021 Jun 7;4:692. doi: 10.1038/s42003-021-02223-w (PMC8184830; doi:10.1038/s42003-021-02223-w)
Supplement: Supplementary file 2 — Supplementary Information [file 42003_2021_2223_MOESM2_ESM.pdf]

Supplementary information

Single-cell RNA sequencing of human nail unit defines *RSPO4* onychofibroblasts and *SPINK6* nail epithelium

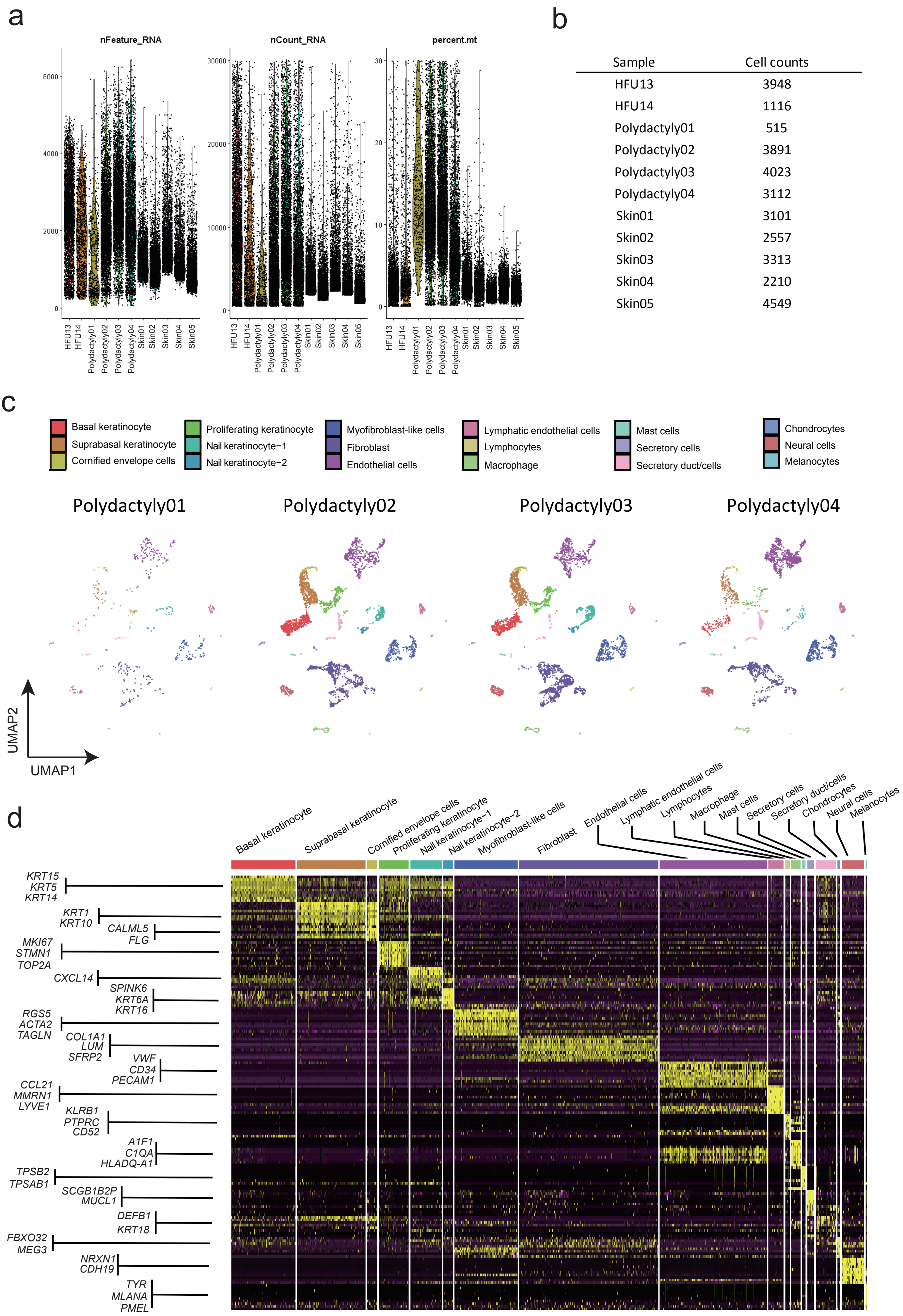

Sample	Cell counts
HFU13	3948
HFU14	1116
Polydactyly01	515
Polydactyly02	3891
Polydactyly03	4023
Polydactyly04	3112
Skin01	3101
Skin02	2557
Skin03	3313
Skin04	2210
Skin05	4549

Supplementary Fig. S1. Summary of scRNAseq dataset used in the analysis.
a, Number of gene feature and count, percent of mitochondria for quality control. **b,** Number of cells in each dataset that passed the quality control. **c,** Individual UMAP plot showing conserved 18 clusters. All clusters were present in each and every individual sample. **d,** Heat map showed discriminative gene sets for each cluster.

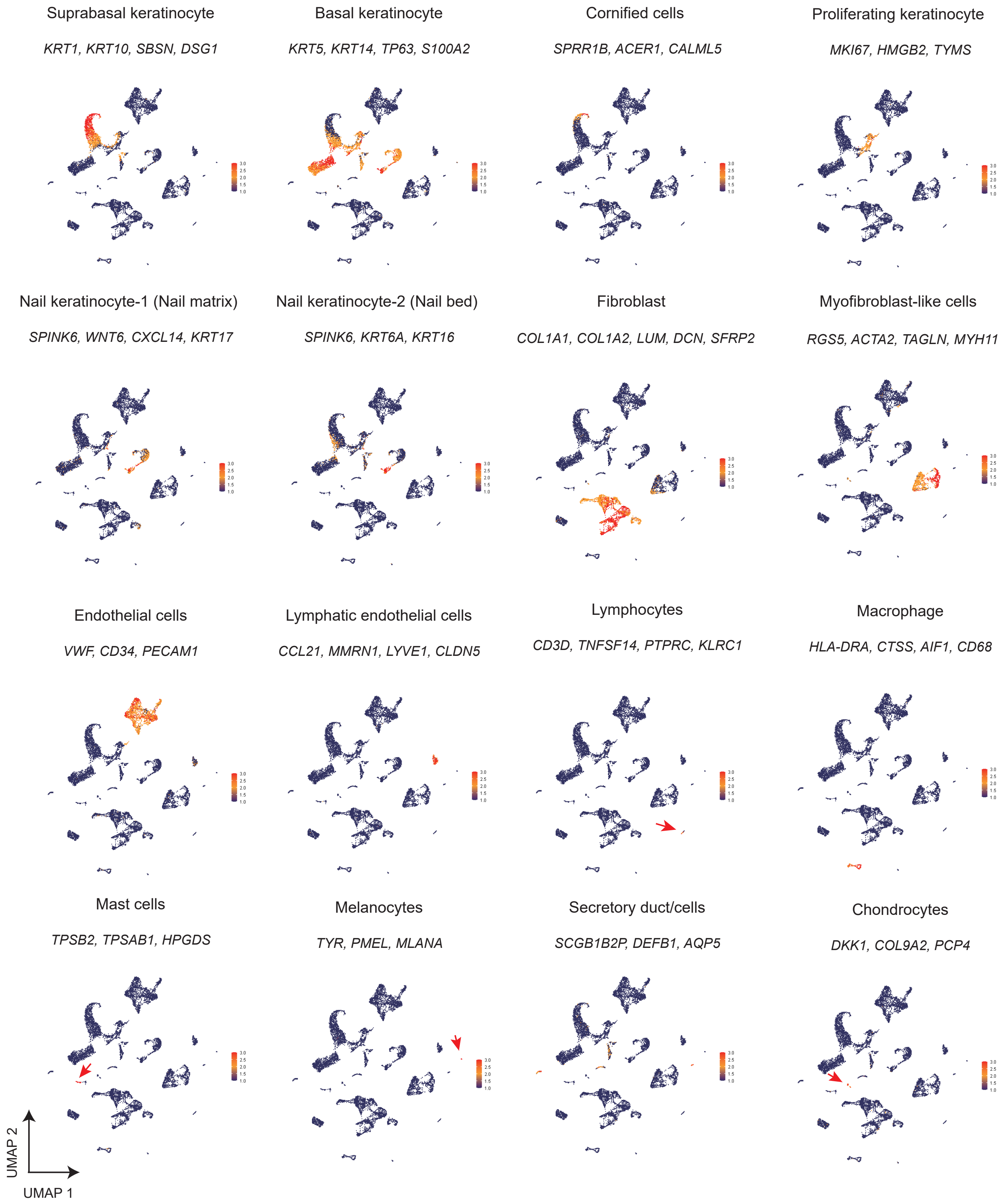

Supplementary Fig. S2. Summary of canonical gene sets used in annotating the clusters. Average expression of 3–5 cell type markers was projected on the UMAP plot. Red indicates maximum gene expression, while blue indicates low or no expression of a particular set of genes in log-normalized UMI counts.

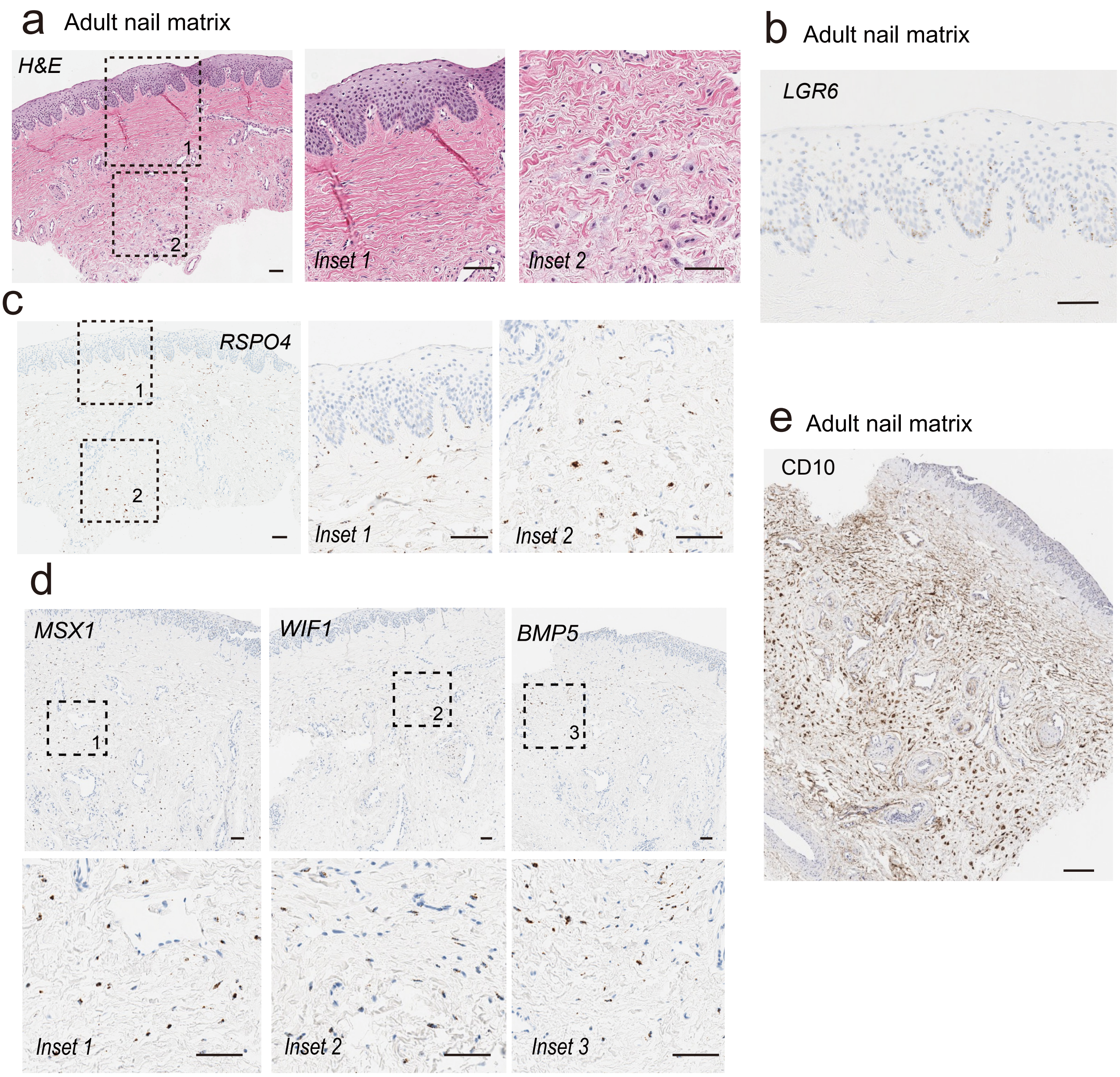

Supplementary Fig. S3: Histological, RNA ISH and immunohistochemical images of adult nail matrix biopsy tissue. a, Hematoxylin and eosin staining showed round cells with prominent nuclei located in the onychomatricodermis[13]. Scale bar = 50 μm. **b,** ISH with *LGR6* probe demonstrated expression of *LGR6* in the basal layer of nail matrix. Scale bar = 50 μm. **c**, ISH demonstrated expression of *RSPO4* in the mesenchyme beneath the nail matrix epithelium including the nuclei of round cells located in the onychomatricodermis. Scale bar = 50 μm. **d**, ISH showed *MSX1*, *WIF1* and *BMP5* expression mainly in the onychomatricodermis (with magnified view indicated by black box). Scale bar = 50 μm. **e**, Immunohistochemical staining of CD10 in adult nail matrix. Scale bar = 200 μm. ISH, in situ RNA hybridization.

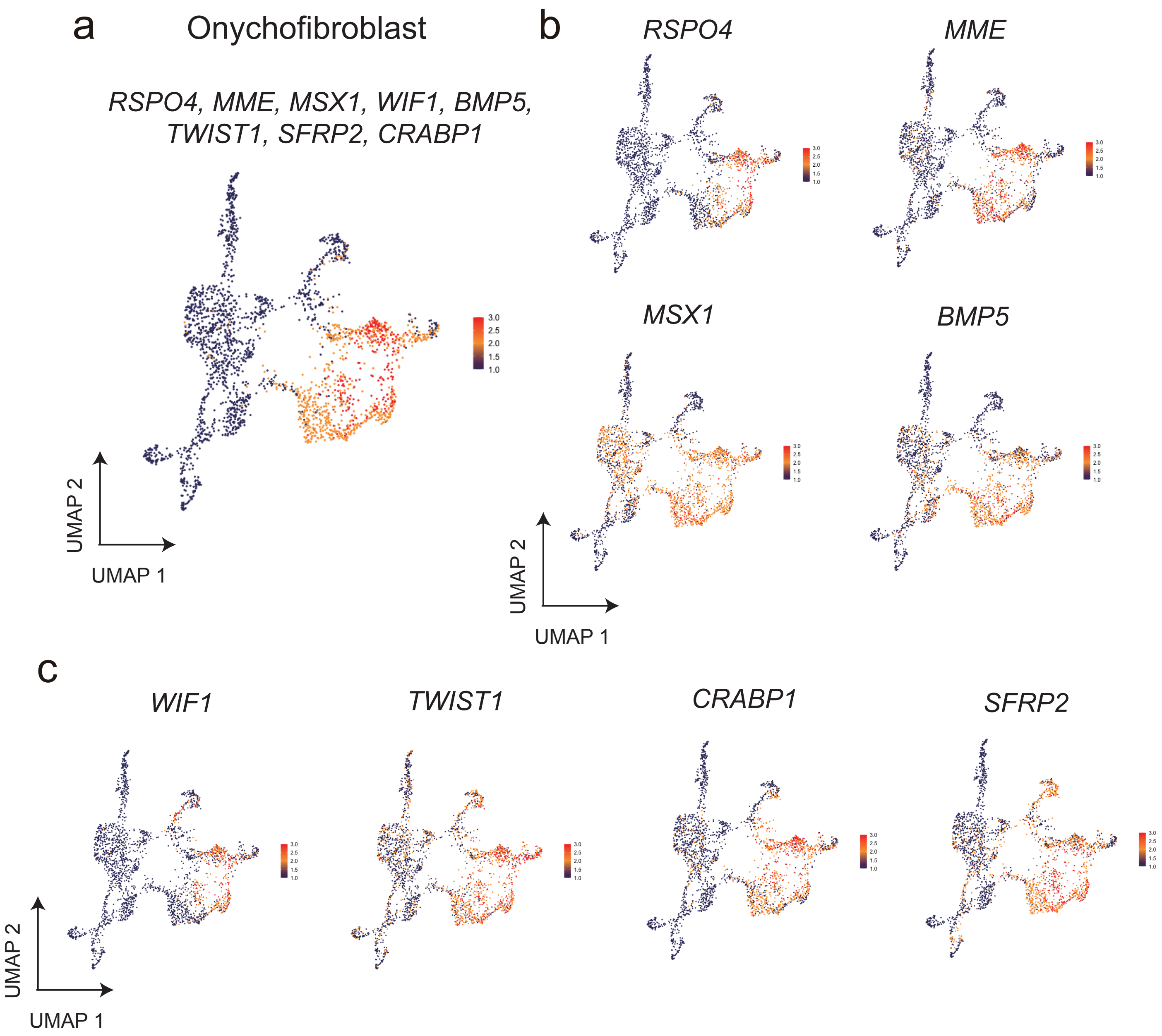

Supplementary Fig. S4. Heterogeneity of onychofibroblasts. a. Gene expression score of onychofibroblast-specific expressed genes. b-c. Feature plots of *RSPO4*, *MME, MSX1, BMP5, WIF1, TWIST1, CRABP1,* and *SFRP2*

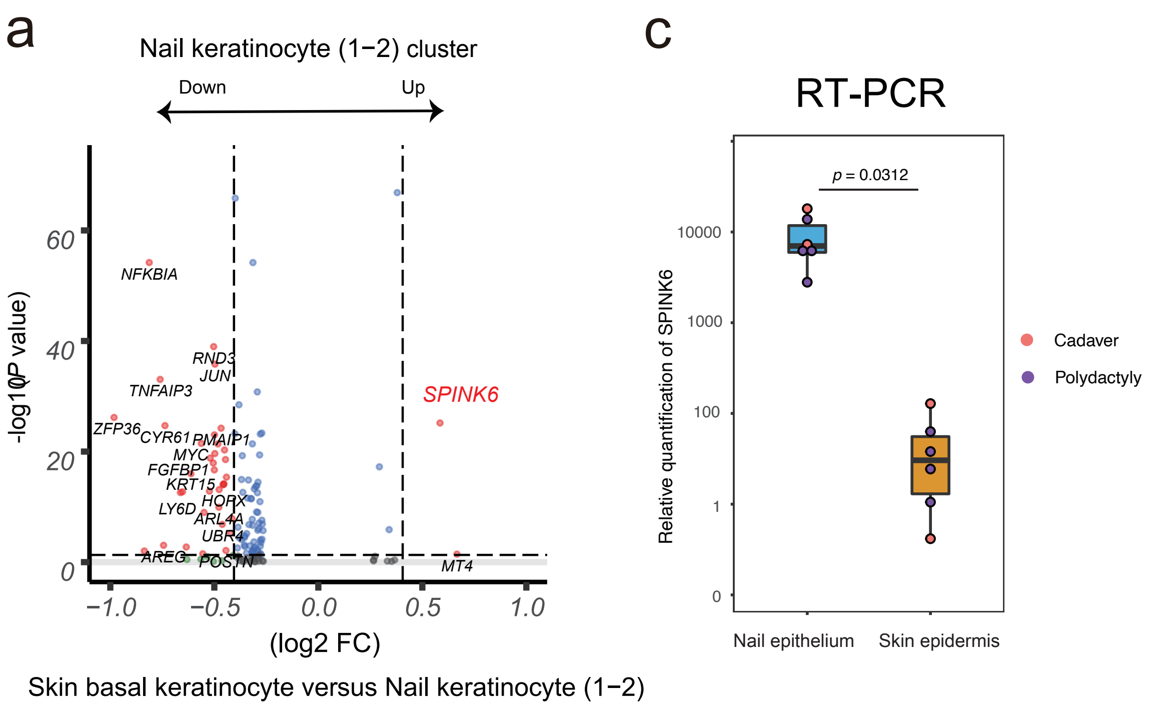

b Microarray

Gene	Sample	Skin epidermis	Nail epithelium	Nail epithelium/Skin epidermis
SPINK6	Test1	81.11	25034.71	308.65
SPINK6	Test2	8.80	27341.68	3110.05

Supplementary Fig. S5. *SPINK6* is highly expressed in nail keratinocytes. a, scRNAseq with polydactyly samples showed distinctively expressed *SPINK6* in nail keratinocyte compared to skin basal keratinocyte. b, Dispase dissociated nail epithelium showed increased expression of *SPINK6* in nail epithelium compared to skin epidermis. c, RT-PCR result from six independent experiment confirmed SPINK6 overexpression in nail epithelium. Four samples were harvested from polydactyly donor and 2 samples were sampled from cadaveric donor. The *p* value is calculated using a paired Wilcoxon test. FC, fold change; scRNA-seq, single-cell RNA sequencing.

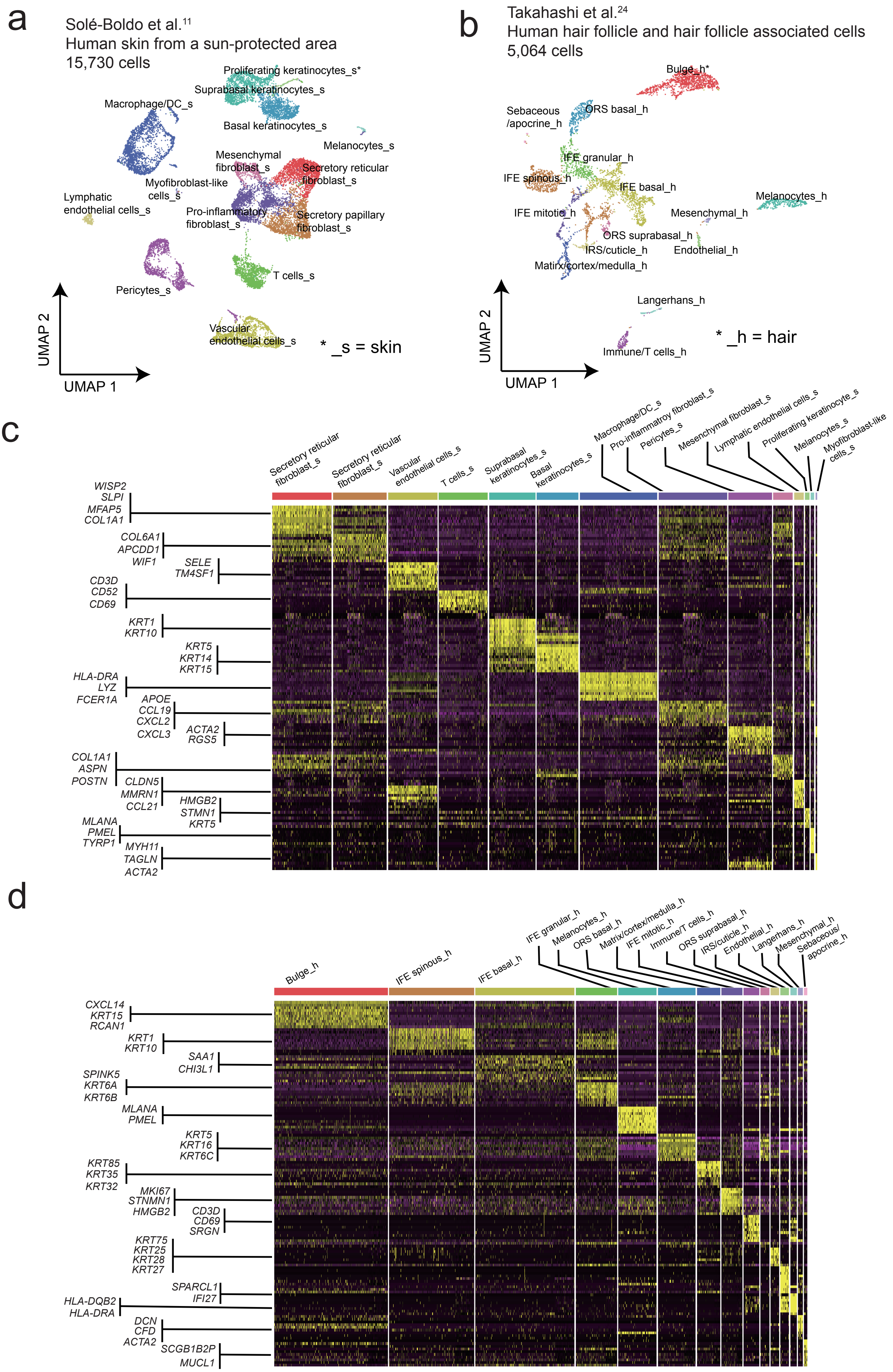
a
Solé-Boldo et al.[11]
Human skin from a sun-protected area
15,730 cells
Proliferating keratinocytes_s*
Suprabasal keratinocytes_s
Macrophage/DC_s
Basal keratinocytes_s
Melanocytes_s
Mesenchymal fibroblast_s
Secretory reticular fibroblast_s
Myofibroblast-like cells_s
Lymphatic endothelial cells_s
Pro-inflammatory fibroblast_s
Secretory papillary fibroblast_s
T cells_s
Pericytes_s
Vascular endothelial cells_s
* _s = skin
UMAP 2
UMAP 1
b
Takahashi et al.[24]
Human hair follicle and hair follicle associated cells
5,064 cells
Bulge_h*
Sebaceous /apocrine_h
ORS basal_h
IFE granular_h
IFE spinous_h
IFE basal_h
Melanocytes_h
IFE mitotic_h
Mesenchymal_h
ORS suprabasal_h
IRS/cuticle_h
Endothelial_h
Matirx/cortex/medulla_h
Langerhans_h
Immune/T cells_h
* _h = hair
UMAP 2
UMAP 1
c
Secretory reticular fibroblast_s
Secretory reticular fibroblast_s
Vascular endothelial cells_s
T cells_s
Suprabasal keratinocytes_s
Basal keratinocytes_s
Macrophage/DC_s
Pro-inflammatroy fibroblast_s
Pericytes_s
Mesenchymal fibroblast_s
Lymphatic endothelial cells_s
Proliferating keratinocyte_s
Melanocytes_s
Myofibroblast-like cells_s
WISP2
SLPI
MFAP5
COL1A1
COL6A1
APCDD1
WIF1
SELE
TM4SF1
CD3D
CD52
CD69
KRT1
KRT10
KRT5
KRT14
KRT15
HLA-DRA
LYZ
FCER1A
APOE
CCL19
CXCL2
CXCL3
ACTA2
RGS5
COL1A1
ASPN
POSTN
CLDN5
MMRN1
CCL21
HMGB2
STMN1
KRT5
MLANA
PMEL
TYRP1
MYH11
TAGLN
ACTA2
d
Bulge_h
IFE spinous_h
IFE basal_h
IFE granular_h
Melanocytes_h
ORS basal_h
Matrix/cortex/medulla_h
IFE mitotic_h
Immune/T cells_h
ORS suprabasal_h
IRS/cuticle_h
Endothelial_h
Langerhans_h
Mesenchymal_h
Sebaceous/ apocrine_h
CXCL14
KRT15
RCAN1
KRT1
KRT10
SAA1
CHI3L1
SPINK5
KRT6A
KRT6B
MLANA
PMEL
KRT5
KRT16
KRT6C
KRT85
KRT35
KRT32
MKI67
STNMN1
HMGB2
CD3D
CD69
SRGN
KRT75
KRT25
KRT28
KRT27
SPARCL1
IFI27
HLA-DQB2
HLA-DRA
DCN
CFD
ACTA2
SCGB1B2P
MUCL1

Supplementary Fig. S6. A UMAP from human skin and hair scRNAseq data. Publicly available human skin and hair scRNAseq data were re-analyzed. **a-d.** Total of 14 and 15 clusters from human skin and hair were re-annotated based on the previous human single cell RNAseq reports. ORS, outer root sheath; IFE, interfollicular epidermis; IRS, inner root sheath; DC, dendritic cells.

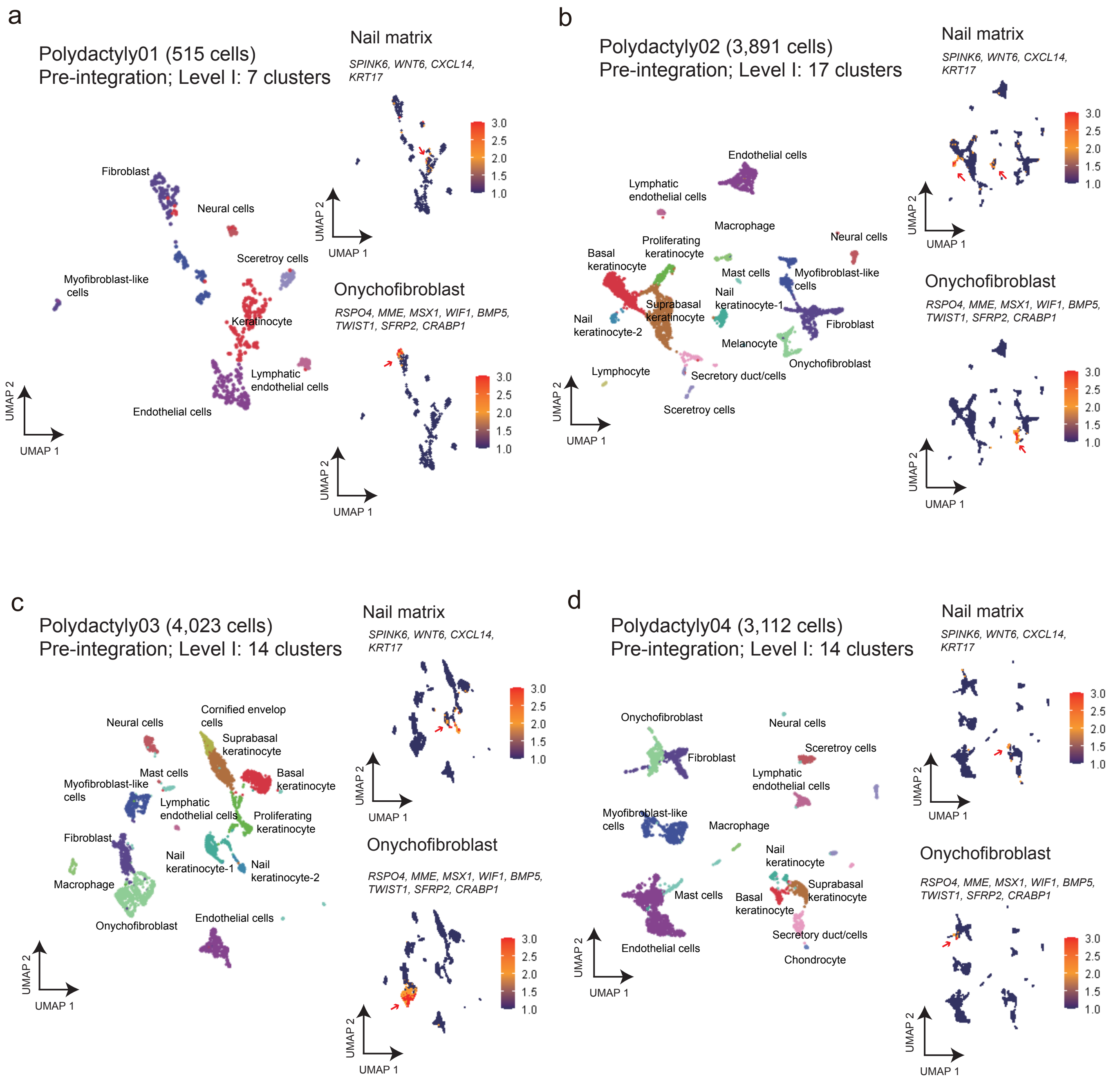

Supplementary Fig. S7. Individual analyses of polydactyly showing the major cell types of the nail unit including onychofibroblast. a-d, UMAP plot resulting from individual sample analyses identified presence of onychofibroblast and nail matrix subpopulations. Average expression of the nail unit specific markers, as determined in **Fig.2b** and **Fig.3b**, projected on the UMAP plot. Red indicates maximum gene expression, while blue indicates low or no expression of a particular set of genes in log-normalized UMI counts.

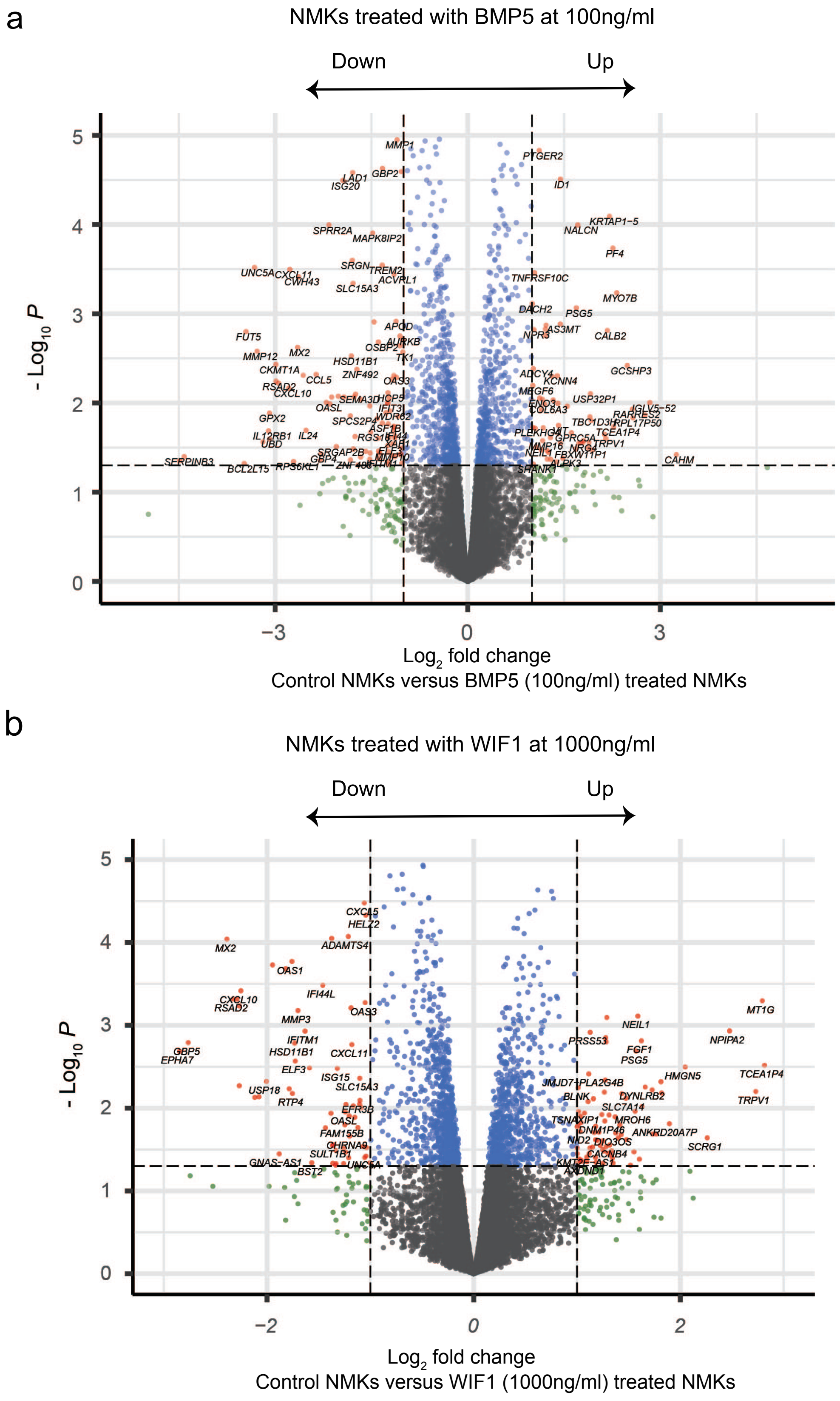

Supplementary Fig. S8. Characterization of DEGs in cultured NMKs after treatment with *BMP5* or *WIF-1*. a. Volcano plot showing DEGs between BMP5 (100ng/ml) treated and control NMKs. A total of 181 DEGs were identified, including ID1. b. Volcano plot depicting DEGs for WIF-1 (1000ng/ml) treated and control NMKs. A total of 153 DEGs were identified.

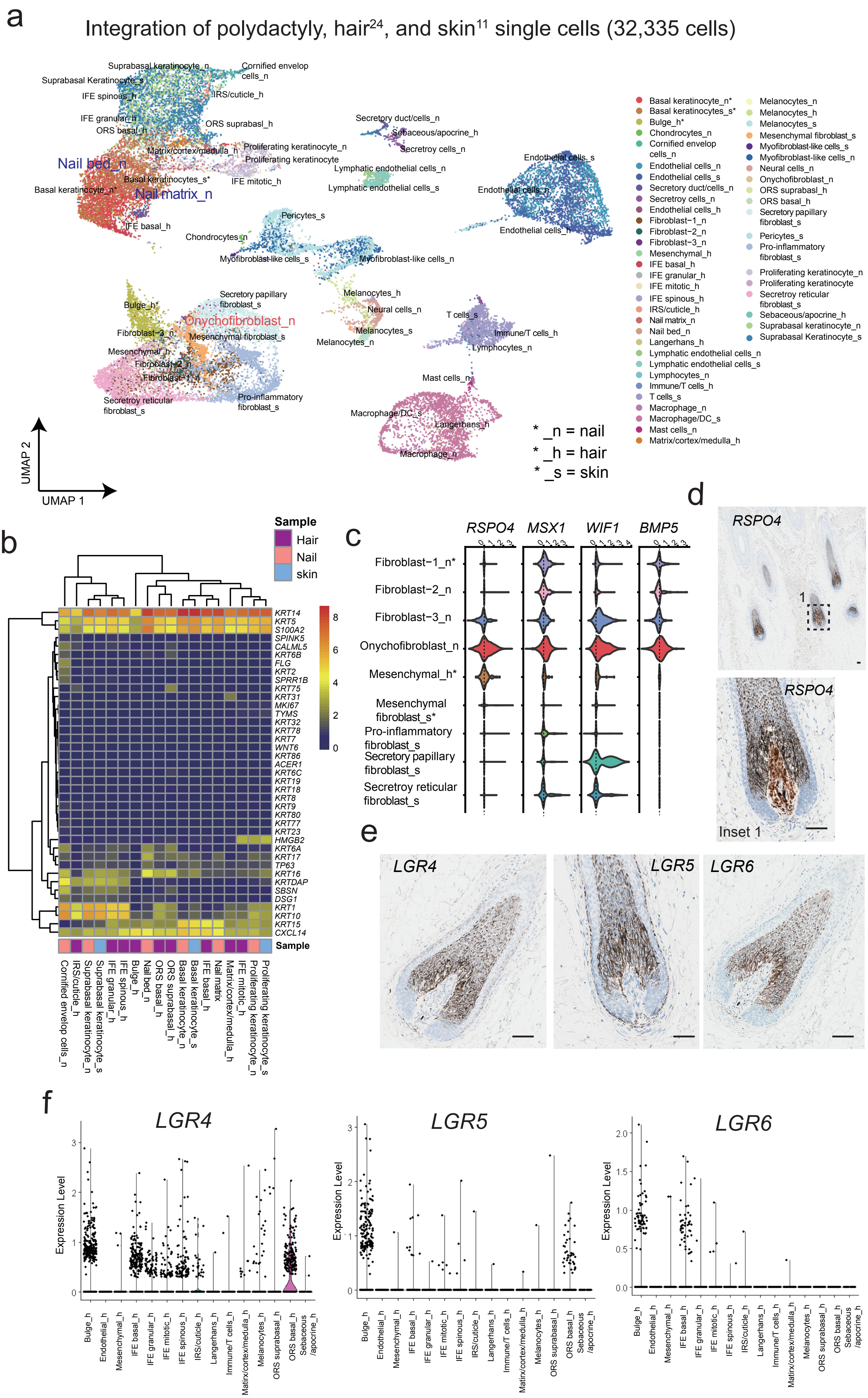
a
Integration of polydactyly, hair24, and skin11 single cells (32,335 cells)
Nail bed_n
Nail matrix_n
Onychofibroblast_n
UMAP 1
UMAP 2
* _n = nail
* _h = hair
* _s = skin
Basal keratinocyte_n*
Basal keratinocytes_s*
Bulge_h*
Chondrocytes_n
Cornified envelop cells_n
Endothelial cells_n
Endothelial cells_s
Secretory duct/cells_n
Secretroy cells_n
Endothelial cells_h
Fibroblast−1_n
Fibroblast−2_n
Fibroblast−3_n
Mesenchymal_h
IFE basal_h
IFE granular_h
IFE mitotic_h
IFE spinous_h
IRS/cuticle_h
Nail matrix_n
Nail bed_n
Langerhans_h
Lymphatic endothelial cells_n
Lymphatic endothelial cells_s
Lymphocytes_n
Immune/T cells_h
T cells_s
Macrophage_n
Macrophage/DC_s
Mast cells_n
Matrix/cortex/medulla_h
Melanocytes_n
Melanocytes_h
Melanocytes_s
Mesenchymal fibroblast_s
Myofibroblast-like cells_s
Myofibroblast-like cells_n
Neural cells_n
Onychofibroblast_n
ORS suprabasl_h
ORS basal_h
Secretory papillary fibroblast_s
Pericytes_s
Pro-inflammatory fibroblast_s
Proliferating keratinocyte_n
Proliferating keratinocyte
Secretroy reticular fibroblast_s
Sebaceous/apocrine_h
Suprabasal keratinocyte_n
Suprabasal Keratinocyte_s
b
Sample
Hair
Nail
skin
KRT14
KRT5
S100A2
SPINK5
CALML5
KRT6B
FLG
KRT2
SPRR1B
KRT75
KRT31
MKI67
TYMS
KRT32
KRT78
KRT7
WNT6
KRT86
ACER1
KRT6C
KRT19
KRT18
KRT8
KRT9
KRT80
KRT77
KRT23
HMGB2
KRT6A
KRT17
TP63
KRT16
KRTDAP
SBSN
DSG1
KRT1
KRT10
KRT15
CXCL14
Cornified envelop cells_n
IRS/cuticle_h
Suprabasal keratinocyte_n
Suprabasal keratinocyte_s
IFE granular_h
IFE spinous_h
Bulge_h
Nail bed_n
ORS basal_h
ORS suprabasal_h
Basal keratinocyte_n
Basal keratinocyte_s
IFE basal_h
Nail matrix
Matrix/cortex/medulla_h
IFE mitotic_h
Proliferating keratinocyte_n
Proliferating keratinocyte_s
c
RSPO4
MSX1
WIF1
BMP5
Fibroblast−1_n*
Fibroblast−2_n
Fibroblast−3_n
Onychofibroblast_n
Mesenchymal_h*
Mesenchymal fibroblast_s*
Pro-inflammatory fibroblast_s
Secretory papillary fibroblast_s
Secretroy reticular fibroblast_s
d
RSPO4
Inset 1
e
LGR4
LGR5
LGR6
f
LGR4
LGR5
LGR6
Expression Level
Bulge_h
Endothelial_h
Mesenchymal_h
IFE basal_h
IFE granular_h
IFE mitotic_h
IFE spinous_h
IRS/cuticle_h
Langerhans_h
Immune/T cells_h
Matrix/cortex/medulla_h
Melanocytes_h
ORS suprabasal_h
ORS basal_h
Sebaceous /apocrine_h

Supplementary Fig. S9. A UMAP from integrated polydactyly, human hair and skin scRNAseq data and *RSPO4* and *LGR4-6* expression in hair follicle. a, Nail-specific keratinocytes are located inside of basal keratinocyte cluster. Nail-specific fibroblasts are located near the mesenchymal fibroblast cluster. **b,** Clustered heatmap of keratinocyte associated gene expressed in nail, hair and skin keratinocytes. **c,** Violin plot for nail, hair and skin fibroblasts. **d,** *RSPO4* is specifically expressed in hair mesenchyme. ISH showed strong *RSPO4* signal in follicular dermal papilla. **e,** *LGR5* expression is found at closest proximity to the *RSPO4* expressing follicular dermal papilla, compared to the other two remaining *RSPO4* receptors, *LGR4* and *LGR6*. **f,** Violin plots for *LGR4,5,* and *6* among all clusters annotated in hair scRNAseq data. Scale bar = 50 μm.

Supplementary Table 1. The summary of read counts and UMI counts for each polydactyly samples.

		Polydactyly 01	Polydacytly 02	Polydacytly 03	Polydacytly 04
Cells	Estimated Number of Cells	1,276	5,613	5,633	4,257
	Fraction Reads in Cells	21.60%	92.00%	91.80%	88.20%
	Mean Reads per Cell	91,044	34,092	33,821	43,181
	Median Genes per Cell	268	1,762	2,305	2,057
	Total Genes Detected	18,325	23,571	23,756	23,524
	Median UMI Counts per Cell	1,367	5,046	7,205	5,578
Sequencing	Number of Reads	116,172,306	191,355,721	190,512,364	183,820,787
	Valid Barcodes	97.00%	96.20%	97.80%	97.70%
	Sequencing Saturation	70.50%	50.80%	44.90%	54.90%
	Q30 Bases in Barcode	97.70%	95.90%	96.50%	97.00%
	Q30 Bases in RNA Read	92.70%	95.00%	88.30%	89.20%
	Q30 Bases in Sample Index	97.40%	95.50%	95.40%	96.40%
	Q30 Bases in UMI	98.10%	94.20%	96.70%	97.10%
Mapping	Reads Mapped to Genome	95.40%	97.40%	96.80%	96.80%
	Reads Mapped Confidently to Genome	84.10%	87.90%	89.80%	90.80%
	Reads Mapped Confidently to Intergenic Regions	30.00%	8.00%	6.90%	6.70%
	Reads Mapped Confidently to Intronic Regions	4.60%	17.40%	17.90%	20.00%
	Reads Mapped Confidently to Exonic Regions	49.50%	62.40%	65.00%	64.10%
	Reads Mapped Confidently to Transcriptome	48.00%	58.70%	61.90%	60.90%
	Reads Mapped Antisense to Gene	0.70%	1.60%	1.10%	1.20%

Supplementary Table 2. Clinical charactericstics of publicly available cohorts.

Sample ID	Gender	Age	Type	Anatomical location of sampling	Cell counts
Skin 01	Male	25	Whole skin	Inguinoiliac region	3,101
Skin 02	Male	27	Whole skin	Inguinoiliac region	2,557
Skin 03	Male	53	Whole skin	Inguinoiliac region	3,313
Skin 04	Male	70	Whole skin	Inguinoiliac region	2,210
Skin 05	Male	69	Whole skin	Inguinoiliac region	4,549
HFU 13	NA	NA	Hair graft	Scalp	3,948
HFU 14	NA	NA	Hair graft	Scalp	1,116

Supplementary Table 3. The differentially expressed genes of Rspo4 induced transcriptomic changes in the nail matrix keratinocytes.

Gene	logFC	Fold change	logCPM	CPM	F	*P*-value
KRTAP2-3	1.7873	3.4517	1.0808	2.1152	18.5388	0.0004
KRTAP1-5	1.6354	3.1067	-0.1420	0.9063	4.6391	0.0442
OR5P3	1.5498	2.9277	0.2020	1.1503	6.3171	0.0211
LGR6	1.4868	2.8027	0.0029	1.0020	12.3230	0.0023
FOXQ1	1.4785	2.7867	-0.3090	0.8072	12.7581	0.0020
SERPINB2	1.3666	2.5786	6.2612	76.7027	13.4389	0.0016
FGF1	1.3541	2.5563	-0.0432	0.9705	13.2743	0.0017
C6orf52	1.2358	2.3551	-0.3771	0.7700	5.5205	0.0297
HOPX	1.1833	2.2710	-0.2596	0.8353	6.9951	0.0159
ALPK3	1.1689	2.2484	-0.4094	0.7530	5.7681	0.0267
SPRR2D	1.1633	2.2397	1.8808	3.6829	9.4507	0.0062
UCP2	1.1418	2.2066	-0.1632	0.8931	8.1914	0.0099
HBEGF	1.1067	2.1535	4.5049	22.7038	4.9673	0.0380
CLYBL	1.1067	2.1535	-0.1465	0.9034	4.7857	0.0413
TRPV3	1.0816	2.1164	4.6599	25.2792	16.4390	0.0007
SNORD64	1.0595	2.0843	-0.3591	0.7797	8.0425	0.0120
MACROD1	1.0213	2.0298	0.5113	1.4254	6.4364	0.0201
HEY1	1.0170	2.0237	0.9795	1.9718	11.6385	0.0029
MCHR1	-1.0112	0.4961	0.0208	1.0146	7.1475	0.0150
MT1M	-1.0230	0.4921	0.0501	1.0354	5.0316	0.0369
LRRC15	-1.0710	0.4760	0.6036	1.5195	7.3368	0.0139
RNASEH2CP1	-1.0860	0.4711	0.2234	1.1675	7.0772	0.0154
C9orf163	-1.0918	0.4692	-0.2566	0.8371	5.6904	0.0276
FCRLB	-1.0950	0.4681	0.0201	1.0140	8.9640	0.0074
FAM43A	-1.1487	0.4510	1.2872	2.4405	5.5330	0.0295
HSD11B1	-1.2431	0.4225	1.2105	2.3142	7.1560	0.0149
