# Supplementary material for: Single-cell RNA sequencing of human nail unit defines RSPO4 onychofibroblasts and SPINK6 nail epithelium
Source: Commun Biol. 2021 Jun 7;4:692. doi: 10.1038/s42003-021-02223-w (PMC8184830; doi:10.1038/s42003-021-02223-w)
Supplement: Supplementary file 3 — Description of Additional Supplementary Files [file 42003_2021_2223_MOESM3_ESM.pdf]

Description of Additional Supplementary Files

File name: Supplementary Data 1

Description: List of differentially expressed genes in each cell cluster identified by Seurat in polydactyly.

File name: Supplementary Data 2

Description: The RNA sequencing count matrix for the nail matrix keratinocytes treated with RSPO4.
