# Supplementary material for: Single-cell RNA sequencing of human nail unit defines RSPO4 onychofibroblasts and SPINK6 nail epithelium
Source: Commun Biol. 2021 Jun 7;4:692. doi: 10.1038/s42003-021-02223-w (PMC8184830; doi:10.1038/s42003-021-02223-w)
Supplement: Supplementary file 6 — Reporting Summary [file 42003_2021_2223_MOESM6_ESM.pdf]

nature research

Corresponding author(s): Dongyoun Lee

Last updated by author(s): Apr 26, 2021

Reporting Summary

Nature Research wishes to improve the reproducibility of the work that we publish. This form provides structure for consistency and transparency in reporting. For further information on Nature Research policies, see our Editorial Policies and the Editorial Policy Checklist.

Statistics

For all statistical analyses, confirm that the following items are present in the figure legend, table legend, main text, or Methods section.

n/a	Confirmed	
☐	☒	The exact sample size (*n*) for each experimental group/condition, given as a discrete number and unit of measurement
☐	☒	A statement on whether measurements were taken from distinct samples or whether the same sample was measured repeatedly
☐	☒	The statistical test(s) used AND whether they are one- or two-sided<br>*Only common tests should be described solely by name; describe more complex techniques in the Methods section.*
☒	☐	A description of all covariates tested
☐	☒	A description of any assumptions or corrections, such as tests of normality and adjustment for multiple comparisons
☐	☒	A full description of the statistical parameters including central tendency (e.g. means) or other basic estimates (e.g. regression coefficient) AND variation (e.g. standard deviation) or associated estimates of uncertainty (e.g. confidence intervals)
☐	☒	For null hypothesis testing, the test statistic (e.g. *F*, *t*, *r*) with confidence intervals, effect sizes, degrees of freedom and *P* value noted<br>*Give P values as exact values whenever suitable.*
☒	☐	For Bayesian analysis, information on the choice of priors and Markov chain Monte Carlo settings
☒	☐	For hierarchical and complex designs, identification of the appropriate level for tests and full reporting of outcomes
☒	☐	Estimates of effect sizes (e.g. Cohen's *d*, Pearson's *r*), indicating how they were calculated

Our web collection on statistics for biologists contains articles on many of the points above.

Software and code

Policy information about availability of computer code

Data collection	The single-cell data collection pipeline was established using CellRanger (v3.1.0)<br>FASTQ reads were mapped to GRCh38
Data analysis	Single-cell-RNA-seq: Seurat package version 3.1.1. in R vesion 3.6.0 software.<br>Ligand-receptor interaction analysis was done with NichNet ver.1.0.<br>The code fro the analysis used in this article are summarized in Github (http://github.com/SMC-Derma/scRNAseq_NailMatrix).<br>All software/pipelines used are detailed in the Methods section.

For manuscripts utilizing custom algorithms or software that are central to the research but not yet described in published literature, software must be made available to editors and reviewers. We strongly encourage code deposition in a community repository (e.g. GitHub). See the Nature Research guidelines for submitting code & software for further information.

Data

Policy information about availability of data

All manuscripts must include a data availability statement. This statement should provide the following information, where applicable:

- Accession codes, unique identifiers, or web links for publicly available datasets
- A list of figures that have associated raw data
- A description of any restrictions on data availability

The scRNAseq data that support the findings of this study has been deposited in the the Gene Expression Omnibus (GEO) under accession code GSE158970. The scRNA seq data from previously published cohorts are available at the GEO under the following accession number: GSE129611 and GSE130973. Any other data are available from the corresponding author upon reasonable request.

Field-specific reporting

Please select the one below that is the best fit for your research. If you are not sure, read the appropriate sections before making your selection.

[x] Life sciences [ ] Behavioural & social sciences [ ] Ecological, evolutionary & environmental sciences

For a reference copy of the document with all sections, see nature.com/documents/nr-reporting-summary-flat.pdf

Life sciences study design

All studies must disclose on these points even when the disclosure is negative.

Sample size	No sample-size calculation was performed. Four polydactyly samples were used in this article. Single-cell experiments were performed on maximally feasible numbers. We analyzed over 10,000 cells, which is comparable to the sample size of previous scRNA-seq studies, and had the power to detect cell heterogeneity, cell proportion, and gene expression difference.
Data exclusions	We filtered out the following cells using per-established exclusion criteria: low quality cells, doublets, cells with high mitochondria contents.
Replication	All attempts at replication were successful. Each cell type of the nail unit was represented in all samples.
Randomization	We did not necessarily randomized the sample.
Blinding	No blinding was used.

Reporting for specific materials, systems and methods

We require information from authors about some types of materials, experimental systems and methods used in many studies. Here, indicate whether each material, system or method listed is relevant to your study. If you are not sure if a list item applies to your research, read the appropriate section before selecting a response.

Materials & experimental systems

n/a	Involved in the study	
[ ]	[x]	Antibodies
[ ]	[x]	Eukaryotic cell lines
[x]	[ ]	Palaeontology and archaeology
[x]	[ ]	Animals and other organisms
[ ]	[x]	Human research participants
[x]	[ ]	Clinical data
[x]	[ ]	Dual use research of concern

Methods

n/a	Involved in the study	
[x]	[ ]	ChIP-seq
[x]	[ ]	Flow cytometry
[x]	[ ]	MRI-based neuroimaging

Antibodies

Antibodies used	Antibodies used: CD10 (clone 56C6; Novocastra, Newcastle, UK),SPINK6 (ab110830; Abcam, Cambridge, UK), β-catenin (14; Cell Marque, CA, USA) or LEF1 (ab137872; Abcam, Cambridge, UK).
Validation	The manufacturers provided the validation documents of the antibodies. When we performed the experiments, we included positive controls and negative controls. All antibodies are used in previous studies, which references can be found on the supplier websites.

Eukaryotic cell lines

Policy information about cell lines

Cell line source(s)	Primary nail matrix keratinocytes (NMK) were harvested from nail matrix biopsy specimens. All samples were collected with informed consent from the donors and ethics approval from Institutional Review Board (IRB number: SMC 2017-10-137).
Authentication	NMKs were used without any cell line authentication.
Mycoplasma contamination	N/A
Commonly misidentified lines (See ICLAC register)	No commonly misidentified cell lines were used.

Human research participants

Policy information about studies involving human research participants

Population characteristics	All polydactyly patients were around 1 year old (6-12 months).
Recruitment	The patients were recruited from the Department of Orthopedic Surgery at Samsung medical Center, Seoul, Republic of Korea, between 2019 and 2020. Extra-digits from 4 patients (6-12 months) were delivered to the dermatologic clinic immediately after the digit removal. All informed consent was signed before enrollment.
Ethics oversight	All samples were collected under Institutional Review Board (IRB)-approved consent (IRB number: SMC 2017-10-137).

Note that full information on the approval of the study protocol must also be provided in the manuscript.
